# Galactomannan and PCR in the Central Nervous System to Detect Invasive Mold Disease - A Retrospective Analysis in Immunocompromised Children

**DOI:** 10.1038/s41598-019-49426-0

**Published:** 2019-09-10

**Authors:** Thomas Lehrnbecher, Peter Michael Rath, Andishe Attarbaschi, Gunnar Cario, Michaela Döring, Olga Moser, Urs Mücke, Fiona Poyer, Sarah Rieken, Christian Temme, Sebastian Voigt, Andreas H. Groll, Melchior Lauten

**Affiliations:** 1 Pediatric Hematology and Oncology, Hospital for Children and Adolescents, University of Frankfurt, Frankfurt, Germany; 2 Institute of Medical Microbiology, University Hospital Essen, Essen, Germany; 3 St. Anna Children’s Hospital, Medical University of Vienna, Pediatric Hematology and Oncology, Vienna, Austria; 4 Christian Albrechts University Kiel, Department of Pediatrics, Pediatric Hematology and Oncology, Kiel, Germany; 5 University Children’s Hospital Tübingen, Department of Paediatric Haematology and Oncology, Tübingen, Germany; 6 University Hospital Aachen, Division of Pediatric Hematology and Oncology, Aachen, Germany; 7 Hannover Medical School, Department of Paediatric Haematology and Oncology, Hannover, Germany; 8 University of Lübeck, Department of Paediatrics, Paediatric Haematology and Oncology, Lübeck, Germany; 9 University Hospital Essen, Department of Paediatrics III, Essen, Germany; 10 University Hospital Charité Berlin, Department of Paediatric Haematology and Oncology, Berlin, Germany; 11 University Children’s Hospital Münster, Infectious Disease Research Program, Department of Paediatric Haematology and Oncology, Münster, Germany

**Keywords:** Diagnostic markers, Paediatric research

## Abstract

Invasive mold disease (IMD) of the central nervous system (CNS) is a severe infectious complication in immunocompromised patients, but early microbiological diagnosis is difficult. As data on the value of biomarkers in the CNS are scarce, in particular in children, we retrospectively analyzed the performance of galactomannan (GM) and PCR assays in CNS samples of 15 children with proven and probable CNS IMD and of 32 immunocompromised children without fungal infection. Galactomannan in the cerebrospinal fluid (CSF) was assessed in nine of the 15 pediatric patients and was positive in five of them. Polymerase chain reaction (PCR) was performed in eight of the 15 patients and detected nucleic acids from molds in six patients. Galactomannan and PCR in CNS samples were the only positive microbiologic parameter in the CNS in three and two patients, respectively. In four patients, PCR specified the pathogen detected in microscopy. Galactomannan and PCR results remained negative in the CSF of all immunocompromised children without evidence for CNS IMD. Our data suggest that GM and PCR in CNS specimens are valuable additional tools in diagnosing CNS IMD and should be included in the work up of all pediatric patients with suspected mold disease of the CNS.

## Introduction

Invasive mold disease (IMD) of the central nervous system (CNS) is a particularly severe infectious complication in immunocompromised patients, and despite the availability of potent antifungal compounds, mortality rates remain unacceptably high^1–3^. Although *Aspergillus* spp is the most frequent cause of CNS IMD, other pathogens such as *Fusarium* spp or the mucormycetes (e.g., *Rhizopus*, *Mucor*, *Rhizomucor*, *Lichtheimia*) also have to be considered^4^. The difficulty to establish a microbiological diagnosis of CNS IMD might explain that data on their epidemiology are scarce, in particular in the pediatric setting. However, early and adequate diagnosis of CNS IMD has important implications on treatment, as therapeutic strategies of CNS IMD may differ from IMD of other organs with regard to the choice and dosage of the antifungal compound and/or surgery.

In immunocompromised patients, radiological signs of CNS IMD can be subtle, and abnormalities of imaging findings are not specific for CNS IMD^4^. Isolation of the pathogen by culture and microscopy are considered the gold-standard for the definite diagnosis of CNS IMD, but unfortunately, both methods lack sensitivity and often remain negative^5,6^. Fungal antigens [e.g., galactomannan (GM), ß-D-glucan] or fungal nucleic acids detected by polymerase chain reaction (PCR) may serve as biomarkers and are suggested by various guidelines as adjunctive diagnostic tools for confirmation of IMD^7,8^, although these recommendations are based on limited data, in particular in pediatric patients. In contrast to fungal biomarkers assessed in the serum and bronchoalveolar lavage fluid^9–12^, extremely little information has been published on the performance of GM and PCR in CNS samples, and most of these data are derived from adult patients. As the value of a diagnostic tool in children may be different relative to adults^9,13^, we analyzed the performance of GM and PCR assays in CNS samples in a case series of 15 children with and in 32 children without CNS IMD.

## Results

A total of 15 children and adolescents were included in the analysis (Table 1). CNS IMD was proven in eight patients and probable in seven patients. Median age of the five female and ten male patients was 16.3 years (range, 3.1–18.0). The majority of patients received treatment for acute leukemia (n = 12), and six of the patients had undergone allogeneic HSCT prior to the occurrence of CNS IMD (Table 1).

**Table 1:** Results of diagnostic tests in pediatric patients with proven or probable invasive mold disease of the central nervous system.

Pt #	Age/sex	Underl. diagnosis	*Culture CNS*	*Micros*. *CNS*	*GM CNS*	*GM BAL*	*GM blood*	*PCR CNS*	*PCR BAL*	*PCR blood*	*Culture BAL*	*Pathol*. *BAL*	*Pathol*. *other sites*	*infection outside CNS*	*CNS infection*
1	16.6 /m	ALL	negative^a^	hyphae^a^	n.d.	n.d.	7.5	*A*. *fum*.^a^	n.d.	n.d.	n.d.	n.d.	hyphae^d^	proven	proven^g, h^
2	17.1/f	AML (HSCT)	n.d.	hyphae^a^	n.d.	n.d.	0.5	*A*. *fum*.^a^	n.d.	n.d.	n.d.	n.d.	hyphae^e^	proven	proven^g^
3	15.2/f	AML (HSCT)	n.d.	n.d.	n.d.	n.d.	n.d.	*Fusarium* spp^a^	n.d.	*Fusarium* spp	n.d.	n.d.	n.d.	proven	probable^g^
4	17.9 /m	ALL	negative^b^	n.d.	2.7^b^	3.0	2.0	n.d.	*A*. *fum*.	n.d.	negative	n.d.	n.d.	proven	probable^g^
5	3.8/f	ALL	n.d.	hyphae^a^	5.4^b^	n.d.	negative	n.d.	n.d.	n.d.	n.d.	n.d.	n.d.	probable	proven^g, h^
6	3.1 /m	SCI (HSCT)	n.d.	hyphae^a^	n.d.	n.d.	5.2	negative^b^	n.d.	n.d.	n.d.	hyphae	n.d.	proven	proven^h^
7	17.7 /m	ALL (HSCT)	negative^b^	hyphae^a^	n.d.	n.d.	negative	negative^a^	negative	n.d.	n.d.	hyphae	n.d.	proven	proven^g^
8	3.8/f	ALL	n.d.	hyphae^a^	negative^b^	n.d.	0.8	*A*. *fum*.^a^	n.d.	n.d.	n.d.	n.d.	hyphae^f^	proven	proven^g^
9	16.3 /m	ALL	n.d.	n.d.	n.d.	1.2	negative	*A*. *fum*.^b^	n.d.	n.d.	A. fum.	n.d.	n.d.	proven	proven^g, h^
10	10.6 /m	ALL	negative^b^	n.d.	4.5^b^	12.3	5.7	n.d.	n.d.	n.d.	n.d.	n.d.	n.d.	probable	probable^g– i^
11	17.5 /m	CML (HSCT)	negative^b^	n.d.	negative^b^	2.0	negative	n.d.	n.d.	n.d.	n.d.	n.d.	n.d.	probable	probable^g^
12	5.1/f	ALL	negative^c^	hyphae^a^	0.6^b^	n.d.	negative	*A*. *fum*.^b^	n.d.	n.d.	n.d.	n.d.	n.d.	possible	proven^g, h^
13	16.9 /m	ALL	negative^b^	n.d.	negative^b^	9.8	0.6	n.d.	n.d.	n.d.	negative	n.d.	n.d.	probable	probable^g^
14	5.7 /m	ALL	negative^b^	n.d.	negative^b^	7.3	negative	n.d.	n.d.	n.d.	n.d.	n.d.	n.d.	probable	probable ^h, i^
15	18.0 /m	CGD (HSCT)	negative^b^	n.d.	6.4^b^	n.d.	2.5	n.d.	n.d.	n.d.	n.d.	hyphae	n.d.	proven	probable^g, h^

Pt patient; f female; m male; ALL acute lymphoblastic leukemia, AML acute myeloid leukemia; HSCT hematopoietic stem cell transplantation; SCI severe immunodeficiency; CML chronic myeloid leukemia; CGD chronic granulomatous disease; CNS central nervous system; micros microscopy; n.d. not done; CSF cerebrospinal fluid; GM galactomannan; BAL broncho-alveolar lavage; *A*. *fum*. *Aspergillus fumigatus.*

Assay performed in ^a^biopsy, ^b^CSF, or ^c^biopsy and CSF

Pathological sites outside the CNS were ^d^gut, ^e^eye, and ^f^appendix

Imaging findings were ^g^focal lesions, ^h^hemorrhage/infarction, and ^i^meningitis.

Samples obtained from the CNS were cultured in nine patients (CSF alone in seven patients, CSF/biopsy and biopsy in one patient each), and all of them remained negative. Microscopic assessment was performed in seven patients (all biopsies) and revealed hyphal structures in all of them. In three of these patients, culture was performed in parallel and remained negative (CSF/biopsy, CSF, and biopsy in one patient each).

**Figure 1:**
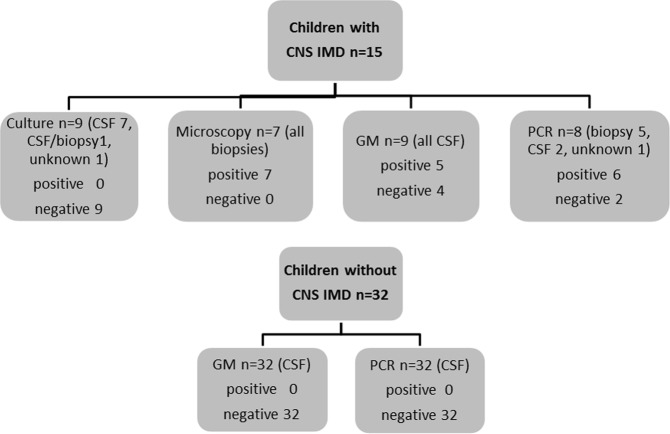
Results of culture, microscopy, and galactomannan (GM) and PCR assays in children with and without proven/probable central nervous system invasive mold disease (CNS IMD).

Galactomannan in the CSF was assessed in the CSF of nine of the 15 pediatric patients, with positive (OD > 0.5) and negative results in five and four patients, respectively (Fig. 1 and Table 1). In four out of the five patients with a positive CSF GM, culture of the CSF was performed in parallel, with negative results in all of them. Microscopic examination and CSF PCR were performed in two and one patient with positive CSF GM and revealed hyphae or detected nucleic acids, respectively. In three out of the four patients with negative GM in the CSF, culture of the CSF was performed and remained negative, whereas in one patient each, microscopic assessment and CSF PCR were performed and were positive. Galactomannan in the CSF was the only positive microbiologic parameter in the CNS of three out of 15 patients with proven and probable CNS IMD (patients #4, 10, 15).

In the five patients with positive GM results in the CSF, GM assessed in the blood and BAL were positive in three and two patients, respectively (assessed in five and two patients, respectively). In the four patients with negative GM in the CNS, GM in blood and BAL were positive in two and three patients, respectively (assessed in four and three patients, respectively).

PCR was performed in eight of the 15 patients (biopsy and CSF as specimen in five and three patients, respectively). PCR detected nucleic acids from *A*. *fumigatus* and *Fusarium* spp in five and in one patient, respectively, and remained negative in two patients (biopsy and CSF as specimen in one patient each) (Fig. 1 and Table 1). In the six patients with positive PCR, other test results from CNS were as follows: negative culture in both patients in whom culture was performed, microscopy positive in all four patients in whom cytology was performed, and GM positive and negative in one patient each (performed in two patients). PCR in the CNS samples were the only positive microbiologic parameter in the CNS of two patients (patients #3 and 9), and specified the pathogen detected in microscopy in four patients (*Aspergillus* in three patients, *Fusarium* in one patient). In the two patients with negative PCR, culture results remained negative (performed in one patient), but microscopy revealed hyphae in both patients in whom microscopy was performed.

In parallel to PCR results from CNS specimen, two patients had PCR results from blood or BAL, respectively: in one patient, both PCR assessments of CNS and blood revealed *Fusarium* spp, and in the other patient, both PCR assays in CNS and BAL remained negative.

Biomarkers were assessed in the CSF of a total of 32 patients without evidence for CNS IMD, leukemic involvement, or inflammation. The median age (range) of these patients was 7 years (1–18), and the underlying diseases were acute leukemia in 25, non-Hodgkin lymphoma in 4, and solid tumor in 3 patients. Galactomannan and PCR remained negative in all of the samples.

## Discussion

Our analysis demonstrates that GM and PCR in CSF or biopsies are useful tools for confirming CNS IMD in immunocompromised children, and that they are helpful to define the causative pathogen. The assessment of GM and PCR was performed in nine and eight out of 15 children with proven/probable CNS IMD, and revealed positive results in five and six patients, respectively (Table 1). In order to avoid major bias, it is necessary to remove patients in whom CNS GM or PCR was used as microbiological criterion to define CNS IMD [in other words: defining a study population by the test to be validated (CSF GM and CSF PCR)]^14^, two out of three CNS GM tests (# 5 and 12) and four out of six CNS PCR (# 1, 2, 8, 12) results indicated CNS IMD, respectively, resulting in a sensitivity of 66% for both tests. On the other hand, all 32 samples in patients without indication of CNS IMD were negative, suggesting a specificity of 100% for both CSF GM and CSF PCR.

Although we recognize that the number of children with CNS IMD included in our analysis was small, one has to consider that early microbiological diagnosis of CNS IMD is extremely difficult, and proven and probable CNS IMD is rare in immunocompromised children^4,15^. Therefore, prospective studies of these severe infections are not feasible, and knowledge will only be based on the analysis of retrospective data. Unfortunately, to date, there are only case reports on diagnostic markers of CNS IMD in immunocompromised children^16–19^, and retrospective analyses in adults include small series of patients with cerebral aspergillosis that evaluated GM and PCR in the CNS, respectively^14,20,21^. However, data regarding diagnostic markers for IFD may not necessarily be transferred from adults to children, as it has been shown for beta-D-glucan and PCR^9,13^, and therefore, the present study is of importance for the pediatric setting. Our data corroborate results of the small adult series, which suggested a sensitivity and specificity of CNS GM of 88% and 96%, and of CNS PCR of 75% and 93%, respectively^14,21^. Notably, the optimal cut-off for positivity of GM in CSF has not been defined to date^7,8^, and has been speculated whether biomarkers such as GM could be important in assessing responses to treatment of CNS IMD^22^. As there are few commercial PCR assays available, most laboratories have developed their in-house methods. It remains unclear whether other PCR methods would have been positive in patient #7, in whom hyphae were detected in CNS biopsy. Currently, various groups such as the European PCR initiative aim to provide standardized protocols suitable for the wide-spread clinical evaluation of PCR testing, and recently proposed the inclusion of PCR assays in the EORTC/MSG definitions^23^. Unfortunately, BDG testing has not been performed in our patient cohort, which is most likely due to the fact that this assay is available only in few laboratories in Germany. Whereas data on the performance of BDG assays in blood are conflicting^24–26^, a recent study suggested the value of this test in detecting CNS IFD^27^.

In conclusion, our analysis suggests that CNS GM and CNS PCR are valuable adjunctive tools in diagnosing CNS IMD and are helpful to further identify the causative pathogen, in particular as conventional diagnostic methods such as culture lack sensitivity. In our analysis, culture of CNS specimens always remained negative, and CNS GM and CNS PCR were the only microbiological tests indicating CNS IMD in five of the patients. We therefore advocate that both CNS GM and CNS PCR should be included in the work up of all pediatric patients with suspected mold infection of the CNS.

## Patients and Methods

For a retrospective study on children and adolescents suffering from CNS IMD, patients were identified by recollection of the local investigators. The clinical details and patients’ outcome have been reported elsewhere as part of a larger cohort^28^. In this analysis, children and adolescents <18 years were included if they (1) received chemotherapy for a malignant disease or underwent allogeneic hematopoietic stem cell transplantation (HSCT), (2) had been diagnosed with proven or probable CNS IMD between 2007 and 2016 and (3) GM and/or PCR was assessed in CNS samples. Pediatric patients with possible CNS IMD were not included in the analysis. Proven CNS IMD was defined as (1) compatible CNS imaging findings^2,4,29^ or macroscopic autopsy findings in combination with (2) a positive microbiologic result of brain biopsy or cerebrospinal fluid^8^. Positive microbiological results included positive culture, microscopic evidence of a mold infection, or the detection of fungal antigens or nucleic acids^2,8,29^. Probable CNS IMD was defined as (1) compatible CNS imaging findings in combination with (2) proven or probable IMD involving a body site outside the CNS^30^. CNS imaging was assessed locally and was not centrally referenced; compatible imaging findings included focal lesions, hemorrhage/infarction and signs of meningitis^4^. Additionally, in patients diagnosed with CNS IMD, the microbiological assessment (e.g., culture, microscopy, galactomannan,and PCR assays) was performed in the local laboratory of each participating center. Due to the retrospective study design, it was impossible to define the exact method of testing (e.g., the specific conditions of an in-house method), and none of the test results has been validated from stored samples, as the storage time might affect the test results^31^.

Galactomannan testing and PCR assays of the control group were performed centrally by one of the authors (PMR), and included immunocompromised children without evidence of CNS IMD, leukemic involvement or inflammatory signs who received a routine diagnostic or therapeutic lumbar puncture. Galactomannan was evaluated using the Platelia^®^ assay (Bio-Rad, Marnes-la-Coquette, France; cut-off 0.5), and *Aspergillus* nucleic acids were detected by the AsperGenius^®^ assay (PathoNostics (Maastricht, Netherlands)^32^. Written informed consent for cancer treatment and indicated supportive care measures and data collection was obtained from all patients and/or their legal guardians. The study was approved by the local research Ethics committee of the University of Lübeck (vote no. 15–301), and all methods were performed in accordance with the relevant guidelines and regulations.
